# Outcomes, Approaches, and Challenges to Developing and Passing a Countywide Mandatory Vaccination Policy: St. Louis County's Experience with Hepatitis A Vaccine for Food Service Personnel

**DOI:** 10.3934/publichealth.2016.1.116

**Published:** 2016-03-15

**Authors:** Terri Rebmann, Kristin D. Wilson, Travis Loux, Ayesha Z. Iqbal, Eleanor B. Peters, Olivia Peavler

**Affiliations:** 1 Institute for Biosecurity, Saint Louis University, College for Public Health & Social Justice, St. Louis, MO, USA,; 2 Department of Health Management and Policy, Saint Louis University, College for Public Health & Social Justice, USA,; 3 Department of Biostatistics, Saint Louis University, College for Public Health & Social Justice, USA,; 4 Center for Clinical Excellence, BJC HealthCare, St. Louis, MO;; 5 St. Louis County Department of Public Health, St. Louis, MO, USA

**Keywords:** Public Health Policy, Mandatory Vaccination, Immunization, Outbreak control

## Abstract

In the early 1990s, St. Louis County had multiple foodservice worker-related hepatitis A outbreaks uncontrolled by standard outbreak interventions. Restaurant interest groups and the general public applied political pressure to local public health officials for more stringent interventions, including a mandatory vaccination policy. Local health departments can enact mandatory vaccination policies, but this has rarely been done. The study objectives were to describe the approach used to pass a mandatory vaccination policy at the local jurisdiction level and illustrate the outcome from this ordinance 15 years later. A case study design was used. In-depth, semi-structured interviews using guided questions were conducted in spring, 2015, with six key informants who had direct knowledge of the mandatory vaccination policy process. Meeting minutes and/or reports were also analyzed. A Poisson distribution analysis was used to calculate the rate of outbreaks before and after mandatory vaccination policy implementation. The policy appears to have reduced the number of hepatitis A outbreaks, lowering the morbidity and economic burden in St. Louis County. The lessons learned by local public health officials in passing a mandatory hepatitis A vaccination policy are important and relevant in today's environment. The experience and lessons learned may assist other local health departments when faced with the potential need for mandatory policies for any vaccine preventable disease.

## 1. Introduction

Despite advances in vaccine development, vaccine preventable diseases continue to pose a risk to United States (U.S.) citizens, especially among certain high-risk groups, such as infants, immunocompromised individuals, and the elderly. Under-vaccinated populations can result in outbreaks when a vaccine preventable disease is introduced through importation after travel or some other type of exposure.[1],[2] Public health professionals, researchers, and policy-makers have used multiple strategies to increase vaccine uptake above herd immunity levels, including education campaigns regarding vaccine safety and efficacy[3],[4], offering free vaccine[5]–[7], and providing vaccine at work sites[5],[6]. However, one of the strongest predictors of vaccine uptake for multiple vaccine types has been implementation of a mandatory vaccination policy.[8]–[10] In the U.S., mandatory vaccination policies often originate from two sources: state law, such as those governing immunization requirements for school admission[11],[12] or the recent passing of California's Senate Bill 277[12]; and healthcare agencies or systems that enact policy covering their employees[13]. Though U.S. city or county health departments have the authority to enact mandatory vaccination policies, this has rarely if ever been done.

This paper is a case study examining the passage of a mandatory hepatitis A vaccine ordinance by the St. Louis County Department of Public Health (STLCoDPH). The local government perspective was adopted for this case study. Unlike California's recent state law[12], this mandatory vaccination policy was formed and implemented at a local level. The purposes of this paper are to: a) describe and reflect upon the approach used to pass a mandatory vaccination policy at the local health department level using a common policy framework to understand the politics, problems, and proposals put forth, and b) examine outcomes from this ordinance 15 years later. Local U.S. public health ordinances frequently only address air quality, food and water safety, sanitation, communicable disease management, and animal/pest control[14]. Information regarding the passage of a mandatory vaccination policy at the local level does not appear in the peer-reviewed literature. Although the actual passage of the mandatory vaccine ordinance was in December 1999, it is relevant and important in today's practice environment to understand the process and lessons learned, as this ordinance is still the only known mandatory vaccination policy passed at the local level. To date, there is very little--if any--documentation in the scientific literature about the process and lessons learned regarding passing of a mandatory vaccine policy at the local level. The case study research questions were as follows:

1. What were the politics, problems, and proposals regarding hepatitis A in St. Louis County that created a policy window of opportunity for passing a mandatory vaccine ordinance? For example, what were the obstacles and enablers in the passing of a mandatory hepatitis A vaccine ordinance by the STLCoDPH for all food service personnel during a time when the Advisory Committee on Immunization Practices (ACIP) did not specifically endorse this action?
2. How can the lessons learned by STLCoDPH be applied to any U.S. city- or county-wide mandatory vaccination policy for vaccine preventable diseases?
3. What impact did passing this county-wide mandatory vaccination policy have on the number and frequency of hepatitis A outbreaks in St. Louis County?

## 2. Materials and methods

### 2.1 Conceptual framework for identifying factors related to problems, politics, and proposals

Kingdon's Agenda-Setting theory is a common policy framework and was used as the conceptual framework for this study.[15] Kingdon's theory describes the flow of three independent “streams” of problems, proposals and politics that, when aligned, create a window of opportunity that facilitates policy change.[15] The “problems” stream involves identification of sustained and uncontrollable hepatitis A outbreaks associated with multiple restaurants and/or foodservice personnel. The “proposals” stream includes the debate around options for addressing the increase in hepatitis A, including the mandatory vaccine proposal. Lastly, the “politics” stream consists of the factors that promoted and inhibited the ability to address the problems and proposed policies around hepatitis A in the food service worker population, such as the political climate, history, and public support.[16] The lessons learned are centered around the “window of opportunity” that was created to obtain the mandatory hepatitis A vaccine policy.[15],[16]

### 2.2 Data analysis for identifying the politics, problems, proposals, and lessons learned

In-depth, semi-structured interviews were conducted with six key informants (five public health officials and one local media contact) who had direct knowledge of and participation in the mandatory hepatitis A vaccination policy process in order to identify the politics, problems, and proposals that created a policy window of opportunity and lessons learned by STLCoDPH. Interviews were conducted in the spring of 2015. The primary author conducted all interviews via telephone over a three-month period. Key informant guiding questions were organized around Kingdon's theory, using a case study approach (see Table 1). Data from the key informant interviews were then narratively and graphically organized to identify the converging policy streams (see Table 2). In addition to qualitative interviews, case study information was obtained from health department employee notes, meeting agendas and minutes, and/or reports written about the ordinance[17]. Data from 1993 through 2015 was obtained from the Missouri Department of Health and Senior Services' communicable disease surveillance system, Websurv.

### 2.3 Data analysis for determining impact of the policy

Impact of the county-wide mandatory vaccination policy was assessed using a Poisson distribution analysis to calculate the rate of outbreaks before and after implementation of the mandatory vaccination policy to determine whether the observed post-intervention (i.e., mandatory vaccination policy) outbreak rate is what would be expected given the pre-intervention outbreak rate. The “before” outbreak rate was calculated from the earliest data available (1993) to policy implementation (1999). The “after” outbreak rate was calculated from the point of policy implementation through the third quarter of 2015. Data was analyzed by quarter (i.e., four data points per calendar year). To determine the population for STLC in 1999, the proxy measure of the census population of STLC in 2000[18], 1,016,315, was used. The Saint Louis University Institutional Review Board approved this study.

**Table 1:** Key Informant Guiding Questions for Semi-Structured Interviews.

What specifically led to the decision to implement a county-wide mandatory hepatitis A vaccination policy for all food services workers/handlers?
What specific actions or processes led to identification for the need for policy change?
In addition to the proposed mandatory policy, what other actions/options were considered as a means of controlling the hepatitis A outbreaks in St. Louis County?
How was the decision made to pursue this ordinance at a local/county level versus on a state level?
Describe the internal process for proposing, drafting, and approving the ordinance, including which group(s) were involved and how it became approved.
What role did the media play in investigating and/or reporting the hepatitis A outbreaks in St. Louis County?
What impact did the media have on proposing the mandatory vaccination policy?
Describe the process used to identify and engage stakeholders to discuss control of the hepatitis A outbreaks and the consideration for a mandatory vaccination policy.
To what extent was there opposition to the proposed mandatory policy? Which group(s) expressed concern over the proposed policy and how was this opposition/concern addressed?
To what extent were local politics involved in proposing this specific ordinance or in investigating the hepatitis A outbreaks in the region?

**Table 2:** Facilitating Factors and Constraints to Passing the Mandatory Vaccination Policy for Food Service Personnel in St. Louis County, MO.

	Facilitating Factors	Constraints
**Problem**	High prevalence of hepatitis A in St. Louis County Hepatitis A outbreaks associated with food service personnel and/or food establishments Outbreaks not controlled using standard measures (e.g., vaccine campaign, restaurant inspections, etc) Outbreak investigation costs	Pre-policy vaccination campaign was unsuccessful Public health resources were constrained by investigating the outbreaks Extensive internal and external communication required Need to establish that a mandatory policy was necessary Lack of a state-wide mandatory vaccination policy or guidance
**Proposals**	Public health officials identified the need for more aggressive intervention(s) to control hepatitis A outbreaks Epidemiological data indicated the way in which St. Louis County was unique from other communities (i.e., linked outbreaks to food establishments and food service personnel) Outbreaks not controlled using aggressive measures (eg, forbidding shared homemade food brought to school) Stakeholder engagement via public forums	Lack of precedence for such a policy ACIP did not specifically endorse routine hepatitis A vaccination for food service personnel Costs and resources needed for implementation and enforcement Concern about vaccine safety among stakeholders Restrictiveness of exemption policy Lack of process/access for administering hepatitis A vaccine to food service personnel in the St. Louis region
**Politics**	A restaurant involved in the hepatitis A outbreak was beloved by the general public, who were upset when the media implied the restaurant was “dirty” Lack of acceptance by general public for the other attempted/proposed control measures Change in tone of media messages from adversarial towards the involved establishments to supportive of finding a solution to the outbreaks Public health officials' willingness to address stakeholder concerns to achieve a compromise to the proposed policy	Public health officials created a sense of urgency related to the outbreaks by sharing epidemiological data Public shaming of restaurants involved in hepatitis A outbreaks via the media Concern among public health officials that the policy might not be acceptable to stakeholders Stakeholder concern that the policy would result in poor food handling practices out of a false sense of security from vaccination

## 3. Results

### 3.1 Politics, Problems and Proposals That Lead to a Policy Window of Opportunity

#### 3.1.1 Problem identification

In the early 1990's, St. Louis County was a high prevalence hepatitis A community, with more than 20.5 cases per 100,000 residents annually. In response, STLCoDPH implemented a hepatitis A vaccine campaign, a strategy recommended by ACIP.[19] Despite the campaign, STLCoDPH experienced three hepatitis A outbreaks between 1993 and 1999. Also notable was that the 1990's St. Louis County outbreaks were unique in that all three occurred at food establishments (two restaurants and one large high school cafeteria) and were epidemiologically linked to infected food handlers.[20],[21] Additionally, facility inspections confirmed environmental contamination associated with person-to-person transmission. This is unlike many U.S. hepatitis A outbreaks, in which only a third of all hepatitis A outbreaks between 1994 and 1999 had a foodborne cause, and of these, less than a quarter involved an infected food handler.[20]

The three St. Louis County outbreaks in the 1990's resulted in a huge financial burden for the STLCoDPH in terms of resources required to respond to these events following historically extant guidelines for prevention and control of restaurant-associated hepatitis A transmission. Personnel time was required to inspect facilities, observe food handling practices, assess personnel for hepatitis A symptoms, conduct contact tracing, and educate food handlers on proper food safety. Additional interventions implemented included forbidding any shared homemade food to be brought to schools and removing community ice bins from restaurants. Medical intervention included provision of Immunoglobulin G (IgG) to more than 7,000 citizens and offer of hepatitis A vaccine. Costs associated with these interventions strained STLCoDPH's limited resources. More importantly, despite strict implementation of interventions, hepatitis A cases in St. Louis County continued to rise.

#### 3.1.2 Politics

Political pressure played a major role in implementation of the mandatory vaccination policy. St. Louis local media were actively involved in reporting outbreak-related events. The media perceived their role as being information conveyors: who was at risk from exposure, which facilities were involved, what type of post-exposure prophylaxis and/or treatment was available, and outbreak prevention. Public health officials and members of the general public perceived media messages to be adversarial; perceptions were that the media blamed the restaurants and/or schools involved in the outbreaks by portraying the facilities as “dirty”. This public shaming proved embarrassing to the facilities involved and reduced business at the restaurants. Early on, negative publicity about the “dirty” restaurants was simply accepted by the general public. However, as more outbreaks occurred and thousands of individuals at multiple restaurants were given IgG for post-exposure follow-up, the general public became angry at the media for tarnishing the reputation of restaurants and schools, and they expressed feelings of being misled by the media about how the infections were occurring. The Health Department Director began working with the media to release more accurate information regarding the outbreaks being a health issue that could be addressed through medical intervention rather than a need to avoid the restaurants or school involved. In addition, restaurant inspection reports and scores (all of which were 95% or greater, indicating strong compliance with food safety regulations and procedures) were shared with the media. When the media messages changed from being adversarial towards the involved establishments to supportive of finding a solution to control the outbreaks, the political landscape shifted to allow for discussion of innovative interventions that might be tried.

The interventions implemented by the STLCoDPH to control the hepatitis A outbreaks, such as eliminating homemade food at schools and removing community ice bins from restaurants, also created political pressure among the general public because parents, restaurant owners, and restaurant patrons were angry about these interventions that were perceived as being draconian. The general public began putting pressure on public health officials to take new steps to control the outbreak. The local Restaurant Association also put pressure on the STLCoDPH to stop the outbreaks, because negative publicity placed a financial burden on local restaurants.

Political pressures peaked when a beloved local restaurant, which many media members and local celebrities/professional athletes patronized often, was implicated in a hepatitis A outbreak. The public did not accept the message that their beloved restaurant was “dirty” or “bad”; community members fought to maintain the positive reputation of the restaurant. The general public grew more vocal about the need to stop the outbreaks, and they were unhappy about the previously implemented interventions that had been ineffective at stopping the outbreak. The hepatitis A outbreak at this restaurant is perceived as the turning point that opened the window of opportunity to expedite the mandatory vaccination policy implementation, because it swung the general public's political support fully to public health officials.

#### 3.1.3 Proposals

Based on the very high hepatitis A case rate in St. Louis County despite interventions, STLCoDPH officials identified the need for aggressive change to halt the current outbreak and prevent future events. The St. Louis County-specific epidemiological data found that all three outbreaks were linked to infected food handlers. Research shows that infected food handlers represent an extremely high risk for the transmission of pathogens to others through food when bare hand contact with ready-to-eat foods and poor hand hygiene are present.[21] As mentioned previously, early on, standard interventions for controlling hepatitis A outbreaks (i.e., investigation, education, administration of IgG, and offering vaccine) were used. However, when months went by and these interventions proved not only costly and resource-intensive, but also ineffective at stopping the outbreaks, the STLCoDPH decided that more robust strategies were warranted to minimize hepatitis A-related morbidity and outbreak costs.

Multiple options were considered to control the outbreak, including closing the restaurants involved and implementing a mandatory vaccination policy. Initially, a mandatory policy was not considered to be a viable option, because there was no such ordinance anywhere in the U.S., and the ACIP did not specifically endorse routine vaccination of food handlers against hepatitis A. There was also fear among STLCoDPH personnel that a mandatory vaccination policy would not be accepted among the Restaurant Association or food handlers, two of the primary stakeholders. It was only after reviewing various options and implementing both standard and more intensive interventions without a noticeable impact on hepatitis A rates that a mandatory vaccination policy was pursued. A mandatory vaccination policy was pursued because it was believed that such a policy would ensure high vaccine uptake, a requirement to prevent future outbreaks, and to assuage the general public and the primary stakeholders who were insisting that aggressive measures be implemented to stop the outbreak.

Many internal and external stakeholders were engaged in order to discuss and pass the mandatory hepatitis A vaccination ordinance. STLCoDPH took the lead in research, development and implementation of the ordinance. Multiple external stakeholders also needed to be engaged in order to pass the mandatory vaccination ordinance. Stakeholders included food handlers, food service facilities/agencies, major food chain and catering services owners, the St. Louis Chapter of the Restaurant Association, the St. Louis County Council, owners of places in which food or drink is prepared for individual sale (e.g. daycare, schools, hospital cafeterias, nursing homes, residential group homes, gas stations, etc.), and members of the general public. The St. Louis County Council held public hearings related to the proposed mandatory policy in each region of the greater St. Louis metropolitan area. Attendance at the public hearings was strong.

**Figure 1:**
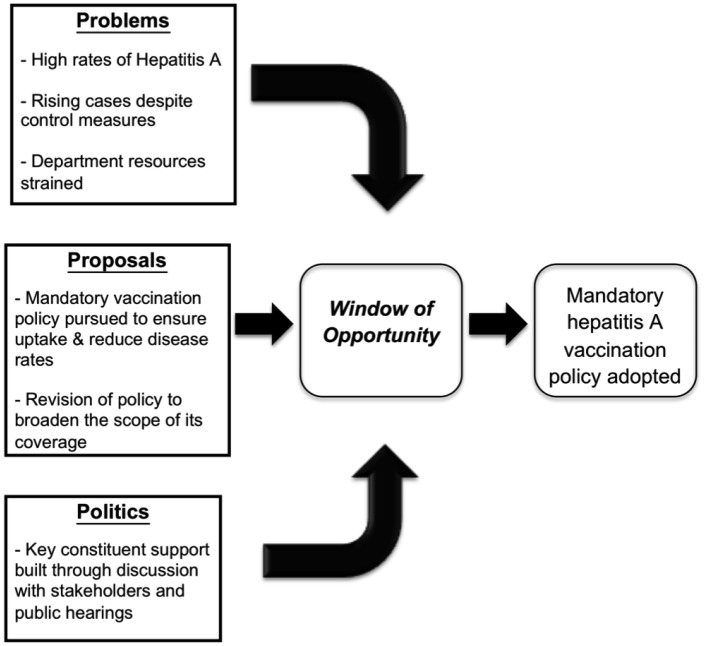
Application of Kingdon's Theory to the Adoption of a Mandatory Hepatitis A Vaccination Policy in St. Louis County.

Multiple obstacles arose during engagement with external stakeholders. Two of the major potential obstacles included a concern among multiple stakeholder groups about vaccine safety and fear of complacency. Some stakeholders expressed worry that vaccinated food handlers would have a false sense of security, which might jeopardize hand hygiene and safe food handling practices. Stakeholders also brought forth the issue of which exemptions would be allowed under the law, and were hesitant to support the policy unless religious exemption was allowed/included. Lastly, stakeholders requested clarification regarding authority and responsibility for enforcing the proposed ordinance. In addition, stakeholders indicated that the lack of access to hepatitis A vaccine in the St. Louis region at that time was a potential major obstacle to implementation of a mandatory vaccination ordinance. Information about stakeholders' concerns raised during public hearings was utilized by the STLCoDPH to clarify and revise the ordinance. After receiving stakeholder input, the STLCoDPH addressed their concerns through presentation of data and discussion of the proposed ordinance at additional public hearings. They also implemented interventions to maximize compliance, such as the STLCoDPH agreeing to provide easy and affordable access to the hepatitis A vaccine through community health centers. After revision, the final bill was forwarded to the St. Louis County Council, the governing body with the authority to pass a mandatory vaccination policy at the County level. Figure 1 depicts the passage and adoption of a mandatory vaccination policy in St. Louis County, including outlining of the politics, problems, and proposals involved.

The initial drafted ordinance required hepatitis A vaccination for all restaurant and supermarket employees and indicated that these staff must receive their first dose of vaccine within 30 days of employment. However, public health officials feared that the hepatitis A outbreaks would continue unless the definition of “food service personnel” was expanded, and they worried that food service personnel would receive the first dose of vaccine, but not follow through on receiving the second/final dose. Therefore, the final approved ordinance indicated that both doses of hepatitis A vaccine would be required within one year of employment for all individuals employed in any capacity in the preparation, handling, or touching of food, utensils, serving items or food preparation surfaces. The ordinance applied to restaurants, cafeterias, school and nursing home kitchens, childcare agencies, residential group homes, sandwich stands, food vending carts, and other such establishments. Three categories were exempt from this law: pregnant women, childcare centers that provide care to 10 or fewer children, and benevolent groups, such as churches hosting a barbeque or other social event where food is served. The law allows for medical and religious exemptions when staff provides written documentation of such. The revisions to the ordinance were acceptable to all stakeholders.

The St. Louis County Council funded the initial purchase of vaccine and the costs associated with hiring additional staff and nurses to provide on- site vaccination at various food establishments. However, food service personnel are now required to pay for their own vaccination unless their employer covers the costs. Compliance with the policy is monitored through the STLCoDPH through routine inspections of all food serving facilities within the jurisdiction. A lack of documented hepatitis A vaccination for all eligible employees is considered a critical ordinance violation and requires that a 10-day follow-up inspection be conducted. If not corrected by the follow-up inspection, a Notice of Violation is issued by STLCoDPH that requires compliance within 10 days. If the establishment remains in violation, they are required to attend an administrative conference with the STLCoDPH to set up a compliance schedule. Continued ordinance violation results in fines and/or establishment closure for 60 days pending violation correction.

#### 3.1.4 Lessons learned

Many important lessons were identified in creating a “window of opportunity” and obtaining the mandatory vaccination policy. The ordinance proposed was novel and somewhat controversial at the time, given that the CDC ACIP does not assert that all U.S. food handlers should receive the hepatitis A vaccine series, instead leaving it up to state and local authorities to regulate. To pass, it required collaboration and extensive communication between the STLCoDPH and key stakeholders, both internal and external, and potential obstacles had to be addressed. Sharing of relevant data, addressing stakeholder concerns, and working with the media were all essential to the passing of this mandatory vaccination policy. The STLCoDPH experience demonstrates that engaging stakeholders and getting them on board a mandatory policy is neither a fast nor easy process, but can be successful. This can be especially complicated when the ACIP does not take a strong stand on a mandatory policy, which was the case with hepatitis A.

### 3.2 Impact of the Mandatory Vaccination Policy

**Figure 2:**
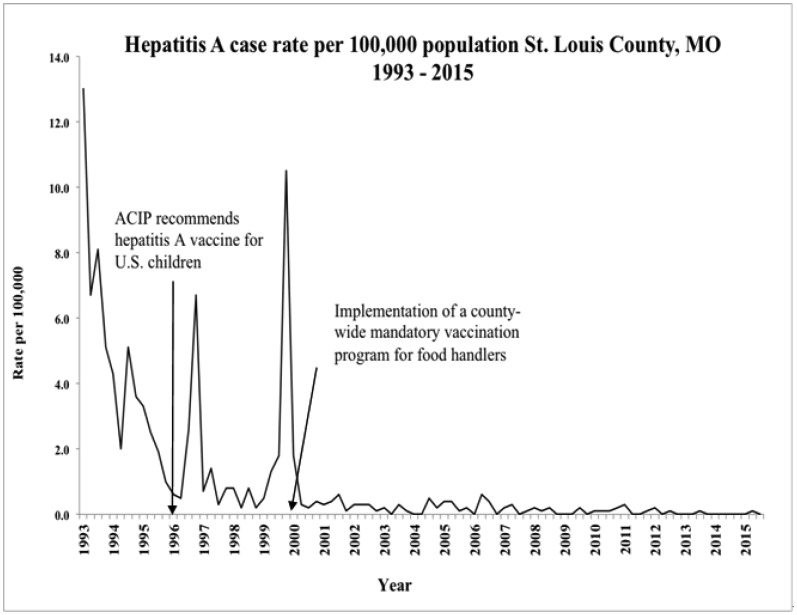
Hepatitis A Case Rates Before Versus After Implementation of a Mandatory Vaccination Policy for Food Handlers in St. Louis County, MO. ACIP = Advisory Committee on Immunization Practices

The ordinance mandating hepatitis A for all food handlers in St. Louis County was passed in December, 1999, and went into effect in 2000. St. Louis County was the first jurisdiction in Missouri to mandate hepatitis A vaccine for food handlers. Figure 2 depicts the STLC hepatitis A case rate between 1993 through the third quarter of 2015. The pre-intervention hepatitis A case rate was 3 per 100,000. After mandatory vaccination policy implementation for food handlers, the average hepatitis A case rate has been 1 per 100,000. The pre-intervention hepatitis A outbreak rate was .40 outbreaks per year (one outbreak every 2 1/2 years) between 1993 and 1999, which includes three years in which the hepatitis A vaccine was available and recommended for U.S. children (i.e., 1996 – 1999). From 2000 through the current time (third quarter of 2015), there have been no hepatitis A outbreaks. Using a Poisson distribution with a rate of .40 (the pre-policy rate), approximately five outbreaks would have been expected to occur between 2000 and 2015, and the probability of zero hepatitis A outbreaks in the post-intervention years would be .005. Interpreting this probability as a p-value gives strong statistical evidence that the rate of hepatitis A outbreaks was lower after the mandatory vaccination policy implementation than before. The number of hepatitis A cases was declining over the pre-intervention years. This may suggest the outbreak rate was also declining; however, testing the post-intervention data (zero outbreaks over 15 years) against an outbreak rate as low as .22 (an outbreak every 4 1/2 years) would still result in statistical significance (a p-value less than .05). Following implementation of this new ordinance, there has been a 10-fold decline in hepatitis A cases and the number of hepatitis A outbreaks has dropped to zero and remained that way for 15 years.

## 4. Discussion

The problem and impact of hepatitis A in St. Louis County was clearly defined, a sense of urgency was created by local public health officials, the general public, and the local Restaurant Association resulting in political pressure to effect change quickly, and the mandatory vaccine proposal created was accepted through an intensive stakeholder engagement process. Although it was eventually passed, implementation of the mandatory hepatitis A vaccination policy for food handlers in St. Louis County was somewhat challenging due to the controversial nature of the ordinance when it was first proposed. When considering a mandatory vaccination policy, context is critical, and experiences will vary by local jurisdictions. However, the obstacles and challenges faced and overcome by the STLCoDPH to create the mandatory vaccination policy for food handlers can serve as a roadmap for other health departments interested in mandatory vaccination policies for any vaccine preventable disease.

It is important to note that mandatory vaccination policies should only be considered when the scientific evidence indicates that such a policy is necessary and can provide a substantial public health impact. Prior to implementation of the mandatory vaccination policy in St. Louis County, the evidence was limited. ACIP's 1999 guidelines[19] (released at the time of St. Louis County's consideration of a new mandatory vaccination policy) discussed the usefulness of administering hepatitis A vaccine during outbreaks, but did not specifically endorse a mandatory vaccination policy for all food handlers. Convergence of the problem, proposal, and politics streams in St. Louis County helped move the ordinance through the policy process, despite the lack of ACIP recommendation. Other jurisdictions can use epidemiological data from their region or published research to provide evidence to their stakeholders that a mandatory vaccination policy is necessary. An example of this translational research is implementation of a mandatory influenza vaccination policy for healthcare personnel. Healthcare system administrators used epidemiological research on the impact of healthcare personnel influenza vaccination on morbidity and mortality of patients, sick leave, and costs related to lost work time[22] to justify implementation of a staff mandatory influenza vaccination policy, despite pushback from some healthcare personnel.[13]

The mandatory vaccination policy for food handlers in St. Louis County appears to be associated with the elimination of hepatitis A outbreaks in the region and with a significant decrease in the hepatitis A case rates from pre- to post-policy implementation. However, given that U.S. hepatitis A rates have decreased dramatically since the availability of hepatitis A vaccine[23] with only sporadic hepatitis A outbreaks nationwide, it cannot be known exactly what role the policy played in this. Furthermore, in today's environment, the need may not exist for a mandatory hepatitis A vaccination policy for food handlers in other parts of the U.S. ACIP recommends that all children receive hepatitis A vaccine, which increases the likelihood that they will be vaccinated. In addition, many unvaccinated individuals in the U.S. have some protection against hepatitis A due to herd immunity. These factors may decrease the likelihood of needing a mandatory hepatitis A vaccine policy in most jurisdictions. However, outbreaks of hepatitis A do continue to occur in the U.S., especially among adult travelers, restaurants or food service businesses, group homes, or other settings in which unvaccinated individuals serve food to others.[20],[24] In high endemic areas or those experiencing outbreaks associated with restaurants or food service personnel, it may be prudent for local public health officials to consider implementing a mandatory vaccination policy for food handlers if standard interventions are not successful at eliminating outbreaks. In that situation, lessons learned from the STLCoDPH ordinance may be beneficial for implementing a similar policy in a new jurisdiction.

The lessons learned by STLCoDPH related to the mandatory hepatitis A vaccine can be generalized to the potential need for other mandatory vaccination policies at a local jurisdiction level. Identifying and understanding the process and obstacles to passing a mandatory vaccination policy is beneficial and relevant in today's environment. The probability of needing to consider a mandatory vaccine policy in local jurisdictions is high, whether for hepatitis A, influenza, pertussis, or other vaccine preventable diseases. Multiple vaccine preventable diseases, such as measles[2] and pertussis[25], are causing outbreaks in the U.S. due to importation from travelers and vaccine uptake rates are lower than necessary to achieve herd immunity. Public health officials may need to consider implementation of new mandatory vaccination policies to halt these outbreaks, such as the 2014 Missouri law requiring all eighth graders to receive a booster of the pertussis vaccine.[26] For those jurisdictions wishing to implement a mandatory vaccination policy, the lessons learned by the STLCoDPH and findings from this study may be useful in determining the best course of action to create a window of opportunity to obtain the mandatory vaccine policy. Local public health does not need to wait for state policy to be implemented; local policy can be developed to address the needs of the community.

### 4.1 Limitations

This case study has limitations. Generalizability is influenced greatly by the context and environment of the problems and politics of local jurisdictions. This case study describes a specific local jurisdiction's experience. The challenges and lessons learned, however, are generalizable and should be considered when planning future work with local ordinance mandatory vaccination programs. There may have been bias recall with key informant interviewees, though information was confirmed through other data sources, such as meeting minutes, to limit this potential bias. Furthermore, hepatitis A rates have dropped nationwide since policies were implemented that encourage children to receive hepatitis A vaccine; therefore, it is not known whether the elimination of hepatitis A outbreaks in St. Louis is due to the mandatory policy or a reflection of nationwide trends. Lastly, because a specific perspective of the local government jurisdiction was adopted for this study, other key stakeholders may provide additional information on the process. However, the lessons learned as reflected upon by the local jurisdiction officials are the most relevant and generalizable to other local jurisdictions. Every effort was made to include all relevant individuals from the local jurisdiction perspective with first-hand knowledge of the policy process.

## 5. Conclusion

Mandatory vaccination policies can be an effective intervention to increase vaccine uptake and reduce morbidity and mortality. However, passing and implementation of such ordinances pose significant hurdles for local public health departments. Lessons learned from the St. Louis County Department of Public Health's mandatory hepatitis A vaccination policy for all food handlers outline effective strategies for passing and implementing such ordinances through the framework of Kingdon's agenda-setting theory. Jurisdictions aiming to decrease morbidity and mortality of vaccine preventable diseases through the increase in vaccine uptake should consider implementing a mandatory vaccination ordinance, following the recommendations set forth in this paper.
